# 4-(3-Methyl-4,5-dihydro-1*H*-benzo[*g*]indazol-1-yl)benzene­sulfonamide

**DOI:** 10.1107/S1600536811033186

**Published:** 2011-08-27

**Authors:** Abdullah M. Asiri, Hassan M. Faidallah, Abdulrahman O. Al-Youbi, Mohamad S. I. T. Makki, Seik Weng Ng

**Affiliations:** a Chemistry Department, Faculty of Science, King Abdulaziz University, PO Box 80203 Jeddah, Saudi Arabia; b Center of Excellence for Advanced Materials Research, King Abdulaziz University, PO Box 80203 Jeddah, Saudi Arabia; c Department of Chemistry, University of Malaya, 50603 Kuala Lumpur, Malaysia

## Abstract

In the title compound, C_18_H_17_N_3_O_2_S, the aromatic ring bearing the sulfamide unit is aligned at 61.65 (1)° with respect to the pyrrole ring; its amino group forms N—H⋯N and N—H⋯O hydrogen bonds to neighboring mol­ecules, generating sheets in the *ac* plane.

## Related literature

For the crystal structure of a pyrrole synthesized using 2-acetyl­tetra­lone as a reactant, see: Portilla *et al.* (2007 ▶).
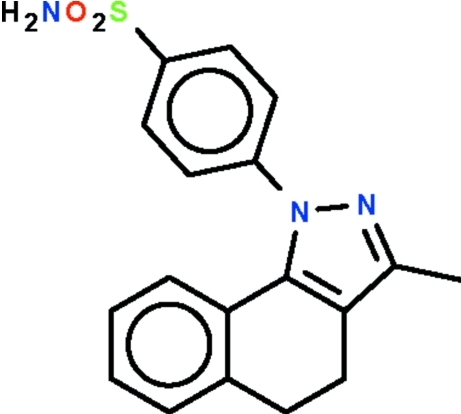


## Experimental

### 

#### Crystal data

C_18_H_17_N_3_O_2_S
*M* *_r_* = 339.41
Monoclinic, 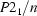

*a* = 4.8838 (1) Å
*b* = 27.3894 (4) Å
*c* = 12.2399 (2) Å
β = 94.738 (1)°
*V* = 1631.67 (5) Å^3^
*Z* = 4
Cu *K*α radiation
μ = 1.89 mm^−1^
*T* = 100 K
0.30 × 0.25 × 0.20 mm

#### Data collection

Agilent SuperNova Dual diffractometer with an Atlas detector
Absorption correction: multi-scan (*CrysAlis PRO*; Agilent, 2010 ▶) *T*_min_ = 0.600, *T*_max_ = 0.703
11808 measured reflections
3255 independent reflections
3166 reflections with *I* > 2σ(*I*)
*R*_int_ = 0.018

#### Refinement

*R*[*F* ^2^ > 2σ(*F* ^2^)] = 0.068
*wR*(*F* ^2^) = 0.184
*S* = 1.11
3255 reflections
226 parameters
14 restraints
H atoms treated by a mixture of independent and constrained refinement
Δρ_max_ = 0.70 e Å^−3^
Δρ_min_ = −0.65 e Å^−3^

### 

Data collection: *CrysAlis PRO* (Agilent, 2010 ▶); cell refinement: *CrysAlis PRO*; data reduction: *CrysAlis PRO*; program(s) used to solve structure: *SHELXS97* (Sheldrick, 2008 ▶); program(s) used to refine structure: *SHELXL97* (Sheldrick, 2008 ▶); molecular graphics: *X-SEED* (Barbour, 2001 ▶); software used to prepare material for publication: *publCIF* (Westrip, 2010 ▶).

## Supplementary Material

Crystal structure: contains datablock(s) global, I. DOI: 10.1107/S1600536811033186/bt5614sup1.cif

Structure factors: contains datablock(s) I. DOI: 10.1107/S1600536811033186/bt5614Isup2.hkl

Supplementary material file. DOI: 10.1107/S1600536811033186/bt5614Isup3.cml

Additional supplementary materials:  crystallographic information; 3D view; checkCIF report

## Figures and Tables

**Table 1:** Hydrogen-bond geometry (Å, °)

*D*—H⋯*A*	*D*—H	H⋯*A*	*D*⋯*A*	*D*—H⋯*A*
N1—H1⋯N3^i^	0.88 (1)	2.05 (1)	2.925 (4)	173 (5)
N1—H2⋯O2^ii^	0.88 (1)	1.95 (2)	2.806 (4)	165 (4)

Symmetry codes: (i) 
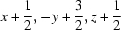
; (ii) 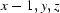.

**Fig. 1.**
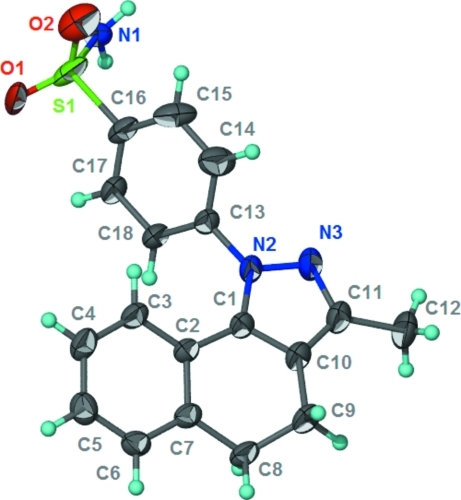
Anisotropic displacement ellipsoid plot (Barbour, 2001) of C18H17N3O2S at the 70% probability level; hydrogen atoms are drawn as spheres of arbitrary radius.

## supplementary crystallographic information

### Comment

Among the wide range of compounds tested for medicinal properties, the compounds having the benzenesulfonamide unit that is grafted to pyrazoles, a class of chemotherapeutically active heterocycles, are expected to exhibit enhanced activity. The ketone, 2-acetyl-tetralone, condenses with a variety of primary amines such as 5-amino-3-methyl-1*H*-pyrazole and 5-amino-3-*tert*-butyl-1*H*-pyrazole (Portilla *et al.*, 2007). However, with 4-hydrazinobenzenesulfamide, the ketone yields a conventional Schiff base that cyclizes to form a pyrazole in a one-pot synthesis. In C_18_H_17_N_3_O_2_S (Scheme I), the benzene and pyrrole rings that are fused to a central cyclohexadiene ring are somewhat twisted owing to the –CH_2_CH_2_– fragment of the cyclohexadiene ring (dihedral angle between benzene and pyrrole rings is 17.3 (2) °. The benzene ring bearing the sulfamide unit is aligned at 61.6 (1) ° with respect to the pyrrole ring; its amino group is hydrogen-bond donor to the acceptor sites of neighboring molecules to generating sheets in the *ac*-plane (Table 1).

### Experimental

2-Acetyl-1-tetralone (1.88 g, 10 mmol) in ethanol (50 ml) condensed with 4-hydrazinobenzenesulfonamide hydrochloride (2.2 g,10 mmol) by heating the reactants for 2 h. The mixture was allowed to cool, and the solid material was collected and recrystallized from ethanol.

### Refinement

Carbon-bound H-atoms were placed in calculated positions [C–H 0.95 to 0.99 Å, *U*_iso_(H) 1.2–1.5*U*_eq_(C)] and were included in the refinement in the riding model approximation. The amino H atoms were located in a difference Fourier, and were refined isotropically with a distance restraint of N–H 0.88±0.01 Å.

As the two oxygen atoms showed somewhat elongated ellipsoids, their anisotropic temperature factors were restrained to be nearly isotropic.

### Crystal data

**Table:**

C_18_H_17_N_3_O_2_S	*F*(000) = 712
*M**_r_* = 339.41	*D*_x_ = 1.382 Mg m^−^^3^
Monoclinic, *P*2_1_/*n*	Cu *K*α radiation, λ = 1.54184 Å
Hall symbol: -P 2yn	Cell parameters from 7385 reflections
*a* = 4.8838 (1) Å	θ = 3.2–74.2°
*b* = 27.3894 (4) Å	µ = 1.89 mm^−^^1^
*c* = 12.2399 (2) Å	*T* = 100 K
β = 94.738 (1)°	Prism, orange brown
*V* = 1631.67 (5) Å^3^	0.30 × 0.25 × 0.20 mm
*Z* = 4	

### Data collection

**Table:**

Agilent SuperNova Dual diffractometer with an Atlas detector	3255 independent reflections
Radiation source: SuperNova (Cu) X-ray Source	3166 reflections with *I* > 2σ(*I*)
Mirror	*R*_int_ = 0.018
Detector resolution: 10.4041 pixels mm^-1^	θ_max_ = 74.4°, θ_min_ = 3.2°
ω scans	*h* = −3→6
Absorption correction: multi-scan (*CrysAlis PRO*; Agilent, 2010)	*k* = −34→33
*T*_min_ = 0.600, *T*_max_ = 0.703	*l* = −14→15
11808 measured reflections	

### Refinement

**Table:**

Refinement on *F*^2^	Primary atom site location: structure-invariant direct methods
Least-squares matrix: full	Secondary atom site location: difference Fourier map
*R*[*F*^2^ > 2σ(*F*^2^)] = 0.068	Hydrogen site location: inferred from neighbouring sites
*wR*(*F*^2^) = 0.184	H atoms treated by a mixture of independent and constrained refinement
*S* = 1.11	*w* = 1/[σ^2^(*F*_o_^2^) + (0.0755*P*)^2^ + 3.2939*P*] where *P* = (*F*_o_^2^ + 2*F*_c_^2^)/3
3255 reflections	(Δ/σ)_max_ = 0.001
226 parameters	Δρ_max_ = 0.70 e Å^−^^3^
14 restraints	Δρ_min_ = −0.65 e Å^−^^3^

### Fractional atomic coordinates and isotropic or equivalent isotropic displacement parameters (Å^2^)

**Table:**

	*x*	*y*	*z*	*U*_iso_*/*U*_eq_	
S1	0.89421 (18)	0.67458 (3)	0.78209 (7)	0.0424 (3)	
O1	0.8478 (8)	0.63001 (10)	0.8387 (2)	0.0718 (11)	
O2	1.1649 (6)	0.69481 (19)	0.7792 (3)	0.0936 (15)	
N1	0.7213 (6)	0.71611 (10)	0.8343 (2)	0.0316 (6)	
N2	0.4538 (6)	0.64587 (9)	0.3184 (2)	0.0325 (6)	
N3	0.2861 (7)	0.68173 (10)	0.2717 (2)	0.0430 (8)	
C1	0.4586 (7)	0.60588 (11)	0.2510 (2)	0.0286 (6)	
C2	0.6045 (6)	0.55949 (10)	0.2675 (2)	0.0260 (6)	
C3	0.8268 (6)	0.55121 (11)	0.3446 (2)	0.0287 (6)	
H3	0.8945	0.5771	0.3909	0.034*	
C4	0.9494 (7)	0.50567 (12)	0.3544 (2)	0.0338 (7)	
H4	1.1012	0.5004	0.4068	0.041*	
C5	0.8501 (7)	0.46774 (12)	0.2875 (3)	0.0348 (7)	
H5	0.9295	0.4361	0.2957	0.042*	
C6	0.6338 (7)	0.47599 (11)	0.2082 (2)	0.0305 (7)	
H6	0.5698	0.4500	0.1616	0.037*	
C7	0.5095 (6)	0.52151 (11)	0.1959 (2)	0.0260 (6)	
C8	0.2724 (7)	0.53032 (12)	0.1096 (2)	0.0312 (7)	
H8A	0.0975	0.5235	0.1423	0.037*	
H8B	0.2875	0.5070	0.0486	0.037*	
C9	0.2622 (8)	0.58217 (12)	0.0634 (2)	0.0373 (8)	
H9A	0.4125	0.5870	0.0149	0.045*	
H9B	0.0850	0.5878	0.0199	0.045*	
C10	0.2932 (7)	0.61727 (12)	0.1578 (2)	0.0340 (7)	
C11	0.1879 (8)	0.66404 (13)	0.1747 (3)	0.0407 (8)	
C12	−0.0164 (11)	0.69262 (15)	0.1018 (4)	0.0586 (12)	
H12A	−0.0683	0.7222	0.1399	0.088*	
H12B	−0.1802	0.6726	0.0836	0.088*	
H12C	0.0660	0.7016	0.0343	0.088*	
C13	0.5657 (7)	0.65231 (11)	0.4290 (2)	0.0293 (7)	
C14	0.7437 (8)	0.69042 (14)	0.4540 (3)	0.0435 (9)	
H14	0.7941	0.7118	0.3979	0.052*	
C15	0.8491 (8)	0.69741 (14)	0.5616 (3)	0.0462 (9)	
H15	0.9728	0.7235	0.5798	0.055*	
C16	0.7714 (7)	0.66571 (12)	0.6426 (3)	0.0316 (7)	
C17	0.5945 (7)	0.62796 (12)	0.6171 (3)	0.0344 (7)	
H17	0.5436	0.6065	0.6729	0.041*	
C18	0.4900 (7)	0.62110 (12)	0.5098 (3)	0.0333 (7)	
H18	0.3664	0.5950	0.4917	0.040*	
H1	0.756 (10)	0.7464 (7)	0.815 (4)	0.061 (14)*	
H2	0.547 (3)	0.7076 (14)	0.829 (3)	0.041 (11)*	

### Atomic displacement parameters (Å^2^)

**Table:**

	*U*^11^	*U*^22^	*U*^33^	*U*^12^	*U*^13^	*U*^23^
S1	0.0376 (5)	0.0539 (6)	0.0321 (5)	0.0161 (4)	−0.0181 (3)	−0.0213 (4)
O1	0.134 (3)	0.0376 (14)	0.0361 (14)	0.0337 (17)	−0.0418 (17)	−0.0086 (11)
O2	0.0354 (16)	0.172 (4)	0.072 (2)	0.001 (2)	−0.0045 (15)	−0.070 (3)
N1	0.0401 (16)	0.0257 (13)	0.0280 (13)	−0.0022 (11)	−0.0041 (11)	−0.0051 (10)
N2	0.0532 (17)	0.0240 (12)	0.0185 (12)	0.0055 (11)	−0.0070 (11)	0.0000 (9)
N3	0.069 (2)	0.0290 (14)	0.0290 (15)	0.0111 (14)	−0.0105 (14)	0.0018 (11)
C1	0.0418 (17)	0.0252 (14)	0.0182 (13)	−0.0001 (12)	−0.0021 (12)	0.0003 (11)
C2	0.0346 (16)	0.0257 (14)	0.0173 (13)	−0.0009 (12)	−0.0006 (11)	−0.0018 (11)
C3	0.0326 (16)	0.0316 (15)	0.0210 (14)	0.0013 (12)	−0.0039 (11)	−0.0035 (11)
C4	0.0394 (18)	0.0405 (17)	0.0208 (14)	0.0080 (14)	−0.0018 (12)	−0.0006 (12)
C5	0.0451 (19)	0.0299 (15)	0.0296 (16)	0.0081 (14)	0.0041 (14)	0.0006 (12)
C6	0.0451 (18)	0.0264 (14)	0.0203 (14)	−0.0047 (13)	0.0041 (12)	−0.0035 (11)
C7	0.0313 (15)	0.0293 (14)	0.0175 (13)	−0.0038 (12)	0.0024 (11)	−0.0023 (11)
C8	0.0371 (17)	0.0366 (16)	0.0189 (14)	−0.0040 (13)	−0.0039 (12)	−0.0048 (12)
C9	0.051 (2)	0.0397 (18)	0.0191 (14)	0.0002 (15)	−0.0066 (13)	−0.0019 (13)
C10	0.0474 (19)	0.0321 (16)	0.0211 (14)	−0.0003 (14)	−0.0058 (13)	0.0024 (12)
C11	0.059 (2)	0.0350 (17)	0.0256 (16)	0.0056 (16)	−0.0105 (15)	0.0034 (13)
C12	0.083 (3)	0.043 (2)	0.044 (2)	0.016 (2)	−0.026 (2)	0.0050 (17)
C13	0.0408 (17)	0.0245 (14)	0.0211 (14)	0.0039 (12)	−0.0056 (12)	−0.0041 (11)
C14	0.057 (2)	0.0407 (19)	0.0329 (18)	−0.0142 (17)	0.0063 (16)	−0.0043 (15)
C15	0.046 (2)	0.049 (2)	0.044 (2)	−0.0169 (17)	0.0021 (16)	−0.0193 (17)
C16	0.0301 (16)	0.0381 (16)	0.0248 (15)	0.0071 (13)	−0.0074 (12)	−0.0104 (12)
C17	0.0456 (19)	0.0330 (16)	0.0227 (15)	0.0008 (14)	−0.0085 (13)	−0.0001 (12)
C18	0.0418 (18)	0.0312 (15)	0.0247 (15)	−0.0050 (13)	−0.0098 (13)	−0.0008 (12)

### Geometric parameters (Å, °)

**Table:**

S1—O1	1.431 (3)	C7—C8	1.521 (4)
S1—O2	1.437 (4)	C8—C9	1.528 (5)
S1—N1	1.582 (3)	C8—H8A	0.9900
S1—C16	1.778 (3)	C8—H8B	0.9900
N1—H1	0.882 (11)	C9—C10	1.502 (4)
N1—H2	0.880 (11)	C9—H9A	0.9900
N2—N3	1.373 (4)	C9—H9B	0.9900
N2—C1	1.372 (4)	C10—C11	1.402 (5)
N2—C13	1.428 (4)	C11—C12	1.503 (5)
N3—C11	1.334 (5)	C12—H12A	0.9800
C1—C10	1.377 (4)	C12—H12B	0.9800
C1—C2	1.463 (4)	C12—H12C	0.9800
C2—C7	1.413 (4)	C13—C14	1.377 (5)
C2—C3	1.398 (4)	C13—C18	1.381 (4)
C3—C4	1.385 (4)	C14—C15	1.387 (5)
C3—H3	0.9500	C14—H14	0.9500
C4—C5	1.385 (5)	C15—C16	1.395 (5)
C4—H4	0.9500	C15—H15	0.9500
C5—C6	1.392 (5)	C16—C17	1.367 (5)
C5—H5	0.9500	C17—C18	1.382 (4)
C6—C7	1.390 (4)	C17—H17	0.9500
C6—H6	0.9500	C18—H18	0.9500
			
O1—S1—O2	121.6 (3)	C9—C8—H8B	108.8
O1—S1—N1	107.85 (18)	H8A—C8—H8B	107.7
O2—S1—N1	104.8 (2)	C10—C9—C8	108.2 (2)
O1—S1—C16	107.07 (16)	C10—C9—H9A	110.1
O2—S1—C16	105.50 (19)	C8—C9—H9A	110.1
N1—S1—C16	109.67 (15)	C10—C9—H9B	110.1
S1—N1—H1	116 (3)	C8—C9—H9B	110.1
S1—N1—H2	109 (3)	H9A—C9—H9B	108.4
H1—N1—H2	116 (4)	C1—C10—C11	106.4 (3)
N3—N2—C1	111.1 (2)	C1—C10—C9	120.5 (3)
N3—N2—C13	118.3 (2)	C11—C10—C9	133.1 (3)
C1—N2—C13	130.1 (3)	N3—C11—C10	110.7 (3)
C11—N3—N2	105.6 (3)	N3—C11—C12	120.8 (3)
N2—C1—C10	106.2 (3)	C10—C11—C12	128.5 (3)
N2—C1—C2	130.0 (3)	C11—C12—H12A	109.5
C10—C1—C2	123.8 (3)	C11—C12—H12B	109.5
C7—C2—C3	119.8 (3)	H12A—C12—H12B	109.5
C7—C2—C1	115.0 (3)	C11—C12—H12C	109.5
C3—C2—C1	125.2 (3)	H12A—C12—H12C	109.5
C4—C3—C2	120.7 (3)	H12B—C12—H12C	109.5
C4—C3—H3	119.6	C14—C13—C18	120.8 (3)
C2—C3—H3	119.6	C14—C13—N2	119.3 (3)
C5—C4—C3	119.8 (3)	C18—C13—N2	119.9 (3)
C5—C4—H4	120.1	C13—C14—C15	119.6 (3)
C3—C4—H4	120.1	C13—C14—H14	120.2
C4—C5—C6	119.9 (3)	C15—C14—H14	120.2
C4—C5—H5	120.1	C14—C15—C16	119.2 (3)
C6—C5—H5	120.1	C14—C15—H15	120.4
C7—C6—C5	121.4 (3)	C16—C15—H15	120.4
C7—C6—H6	119.3	C17—C16—C15	120.8 (3)
C5—C6—H6	119.3	C17—C16—S1	118.7 (3)
C6—C7—C2	118.4 (3)	C15—C16—S1	120.5 (3)
C6—C7—C8	121.2 (3)	C16—C17—C18	119.8 (3)
C2—C7—C8	120.4 (3)	C16—C17—H17	120.1
C7—C8—C9	113.8 (3)	C18—C17—H17	120.1
C7—C8—H8A	108.8	C17—C18—C13	119.8 (3)
C9—C8—H8A	108.8	C17—C18—H18	120.1
C7—C8—H8B	108.8	C13—C18—H18	120.1
			
C1—N2—N3—C11	−0.4 (4)	C8—C9—C10—C1	34.6 (4)
C13—N2—N3—C11	−173.2 (3)	C8—C9—C10—C11	−147.1 (4)
N3—N2—C1—C10	1.3 (4)	N2—N3—C11—C10	−0.7 (4)
C13—N2—C1—C10	173.0 (3)	N2—N3—C11—C12	177.0 (4)
N3—N2—C1—C2	−179.2 (3)	C1—C10—C11—N3	1.5 (4)
C13—N2—C1—C2	−7.5 (6)	C9—C10—C11—N3	−177.0 (4)
N2—C1—C2—C7	163.9 (3)	C1—C10—C11—C12	−176.0 (4)
C10—C1—C2—C7	−16.6 (5)	C9—C10—C11—C12	5.5 (7)
N2—C1—C2—C3	−17.6 (5)	N3—N2—C13—C14	−63.9 (5)
C10—C1—C2—C3	161.8 (3)	C1—N2—C13—C14	124.9 (4)
C7—C2—C3—C4	−2.2 (5)	N3—N2—C13—C18	114.7 (4)
C1—C2—C3—C4	179.4 (3)	C1—N2—C13—C18	−56.5 (5)
C2—C3—C4—C5	−0.4 (5)	C18—C13—C14—C15	0.2 (6)
C3—C4—C5—C6	2.2 (5)	N2—C13—C14—C15	178.8 (3)
C4—C5—C6—C7	−1.5 (5)	C13—C14—C15—C16	−0.3 (6)
C5—C6—C7—C2	−1.1 (5)	C14—C15—C16—C17	0.3 (6)
C5—C6—C7—C8	−179.7 (3)	C14—C15—C16—S1	−178.1 (3)
C3—C2—C7—C6	2.9 (4)	O1—S1—C16—C17	18.0 (3)
C1—C2—C7—C6	−178.5 (3)	O2—S1—C16—C17	148.8 (3)
C3—C2—C7—C8	−178.5 (3)	N1—S1—C16—C17	−98.8 (3)
C1—C2—C7—C8	0.1 (4)	O1—S1—C16—C15	−163.6 (3)
C6—C7—C8—C9	−148.2 (3)	O2—S1—C16—C15	−32.7 (4)
C2—C7—C8—C9	33.2 (4)	N1—S1—C16—C15	79.7 (3)
C7—C8—C9—C10	−48.0 (4)	C15—C16—C17—C18	−0.3 (5)
N2—C1—C10—C11	−1.6 (4)	S1—C16—C17—C18	178.2 (3)
C2—C1—C10—C11	178.8 (3)	C16—C17—C18—C13	0.2 (5)
N2—C1—C10—C9	177.1 (3)	C14—C13—C18—C17	−0.2 (5)
C2—C1—C10—C9	−2.5 (5)	N2—C13—C18—C17	−178.8 (3)

### Hydrogen-bond geometry (Å, °)

**Table:**

*D*—H···*A*	*D*—H	H···*A*	*D*···*A*	*D*—H···*A*
N1—H1···N3^i^	0.88 (1)	2.05 (1)	2.925 (4)	173 (5)
N1—H2···O2^ii^	0.88 (1)	1.95 (2)	2.806 (4)	165 (4)

Symmetry codes: (i) *x*+1/2, −*y*+3/2, *z*+1/2; (ii) *x*−1, *y*, *z*.
